# Developmental and Neurobehavioral Toxicity of Tetrabromobisphenol A Mono(2-hydroxyethyl) Ether (TBBPA-MHEE) in Zebrafish Larvae: Oxidative/Inflammatory Responses and Candidate ErbB-Related Signaling

**DOI:** 10.3390/biology15141120

**Published:** 2026-07-10

**Authors:** Yuhan Deng, Yuqi Zhao, Jiujiu Cao, Tianyu Chen, Ziyu Jiang, Yamin Zhang, Jiannan Chen

**Affiliations:** 1 College of Food and Pharmaceutical Engineering, Nanjing Normal University, Nanjing 210023, China; 2 College of Life Sciences, Nanjing Normal University, Nanjing 210023, China

**Keywords:** tetrabromobisphenol A mono(2-hydroxyethyl) ether (TBBPA-MHEE), zebrafish, neurodevelopmental toxicity, oxidative stress, ErbB signaling pathway, PI3K/Akt/mTOR axis

## Abstract

Tetrabromobisphenol A mono(2-hydroxyethyl) ether (TBBPA-MHEE) is an important byproduct during the production of tetrabromobisphenol A (TBBPA) and its related derivatives. Although it has been detected in aquatic environments, its in vivo developmental toxicity and underlying mechanisms remain poorly understood. In this study, zebrafish were used as a model organism to evaluate the early developmental toxicity, neurobehavioral toxicity, and candidate molecular responses associated with TBBPA-MHEE. The 96 h median lethal concentration (96 h-LC_50_) of TBBPA-MHEE for zebrafish embryos/larvae was 1.684 mg/L. Sublethal nominal exposure concentrations (2, 20, and 200 μg/L) caused developmental abnormalities, including reduced body length, pericardial edema, impaired swim bladder development, and significantly inhibited spontaneous motor activity as well as the response to light–dark transition and mechanical stimulation. Transgenic reporter assays further showed shortened motor neuron projections, reduced brain-region fluorescence in *Tg(gad1b:mCherry)* larvae, and downregulated the expression of neurodevelopment-related genes. Network toxicology analysis suggested that MTOR, SRC, MAPK3, and GSK3B were identified as candidate targets potentially associated with TBBPA-MHEE-induced neurotoxicity, with significant enrichment of the ErbB signaling pathway and possible perturbation of PI3K/Akt/mTOR-related responses. In addition, TBBPA-MHEE exposure increased the accumulation of reactive oxygen species (ROS) in the larval brain and induced inflammatory and apoptotic responses. Quercetin intervention partially alleviated ROS accumulation and inflammation and improved the developmental and motor phenotypes. Collectively, these findings indicate that TBBPA-MHEE induces neurodevelopmental toxicity in zebrafish, possibly associated with altered transcriptional responses related to ErbB signaling and the PI3K/Akt/mTOR axis, accompanied by oxidative stress, inflammatory responses, and apoptosis-related events. Because exposure concentrations were not analytically verified, all treatment levels are reported as nominal concentrations. This study provides experimental evidence for the toxicological assessment and environmental risk evaluation of TBBPA derivative pollutants.

## Simple Summary

This study shows that nominal exposure to TBBPA-MHEE causes developmental abnormalities, motor deficits, and neural injury-related phenotypes in zebrafish larvae. These effects were accompanied by oxidative stress, inflammatory responses, apoptosis-related/cell-death-associated signals, and altered transcription of genes related to ErbB and PI3K/Akt/mTOR signaling. Quercetin partially alleviated oxidative and inflammatory responses and improved selected developmental and behavioral outcomes.

## 1. Introduction

Tetrabromobisphenol A (TBBPA) is among the most widely used brominated flame retardants worldwide [1,2]. In addition to its direct use in printed circuit boards, computers, and other polymer materials, TBBPA is also used as a precursor for synthesizing its derivatives, such as tetrabromobisphenol A bis(2-hydroxyethyl) ether (TBBPA-DHEE) and tetrabromobisphenol A bis(allyl ether) (TBBPA-BAE) [3,4]. TBBPA-MHEE has been identified as a byproduct formed during the production of TBBPA and its related derivatives [5]. With the large-scale manufacturing and continued use of TBBPA-based flame retardants, TBBPA and its derivatives have been detected in various environmental media, including freshwater and soil [6,7], suggesting that TBBPA-MHEE may also pose a potential environmental exposure risk.

Environmental occurrence data further support the need to evaluate this compound. TBBPA-MHEE has been reported in rivers and nearshore surface waters near an e-waste dismantling area in Taizhou, China, with a detection rate of 72.1%, a mean concentration of 2.216 μg/L, and a maximum concentration of 3.46 μg/L [8,9]. Additionally, TBBPA-MHEE has been reported in water bodies associated with industrial discharges in Shouguang, Shandong Province [10]. Moreover, TBBPA derivatives are generally hydrophobic and have potential for bioaccumulation, and they have been detected in various aquatic organisms [5,11,12]. However, due to limitations in the availability of reference standards, analytical sensitivity, and the complexity of environmental samples, the environmental distribution, transformation behavior, and in vivo exposure characteristics of TBBPA-MHEE remain insufficiently elucidated [8,13]. Given the potential for trophic transfer and continuous exposure, the potential ecological and health risks of TBBPA-MHEE warrant further investigation.

Toxicological information on TBBPA-MHEE remains limited. Existing in vitro studies have shown that TBBPA-MHEE can alter lipid metabolism-related processes in models such as 3T3-L1 cells [14,15]. However, its developmental toxicity and organ-specific toxicity in whole organisms remain to be explored. Previous studies have indicated that TBBPA-DHEE, a derivative structurally related to TBBPA-MHEE, induces developmental abnormalities and neurobehavioral deficits in zebrafish larvae [3]. TBBPA-MHEE shares high structural similarity with TBBPA-DHEE [16], and accumulating evidence suggests that neurotoxicity is one of the most prominent toxic effects of TBBPA and its derivatives [17]. These findings suggest that TBBPA-MHEE may also have neurodevelopmental toxicity, which requires further systematic experimental evaluation.

The zebrafish (*Danio rerio*) is a well-established model organism for toxicity testing and is widely used to study the developmental and neurotoxic effects of environmental pollutants, owing to its rapid development, optical transparency during embryonic stages, and suitability for in vivo and high-throughput phenotypic analyses [18,19,20]. Advances in transgenic technology have further enabled direct observation of specific neural phenotypes. For example, *Tg(hb9:eGFP)* and *Tg(gad1b:mCherry)* zebrafish can be used to assess motor neuron development and GABAergic neuron-related signaling, respectively, thereby enhancing the sensitivity and mechanistic resolution of neurotoxicity detection for environmental pollutants [3,21]. Based on these advantages, this study systematically investigated the effects of TBBPA-MHEE on early development, neurobehavioral function, and neural structural phenotypes in zebrafish. Furthermore, network toxicology analysis, gene expression profiling, and quercetin intervention experiments were integrated to explore the underlying mechanisms, aiming to provide a toxicological basis for the ecological risk assessment and environmental management of TBBPA-MHEE and related TBBPA-derived pollutants.

## 2. Materials and Methods

### 2.1. Drugs and Reagents

TBBPA-MHEE (purity ≥ 97%) was synthesized by the laboratory of Jiangsu University. This compound has been used as a reference standard in previous studies for the quantitative detection of this substance in environmental samples, and it meets the relevant analytical standards [8,9]. Trizol and dimethyl sulfoxide (DMSO, purity > 99.7%) were purchased from Sigma-Aldrich Corporation (St. Louis, MO, USA). RT SuperMix for qPCR (+gDNA wiper) for reverse transcription and ChamQ Universal SYBR qPCR Master Mix were purchased from Nanjing Novozymes Bio-technology Co., Ltd. (Nanjing, China).

### 2.2. Zebrafish Lines and Embryo Collection

This study used wild-type AB strain zebrafish, *Tg(hb9:eGFP)* transgenic zebrafish with green fluorescent protein-labeled motor neurons, *Tg(gad1b:mCherry)* transgenic zebrafish with red fluorescent protein-labeled GABAergic neurons, and *Tg(lyz:DsRed)* transgenic zebrafish with red fluorescent protein-labeled neutrophils. All adult fish lines were obtained from the Nanjing Yaoshunyu Biotechnology Co., Ltd. (Nanjing, China) and maintained under laboratory conditions. Adult zebrafish were reared at 27.5–28.5 °C in water with a conductivity of 400–500 μS/cm, pH 7.0–8.0, under a photoperiod of 14 h light/10 h dark. One week prior to the experiments, healthy adult fish were selected and kept in separate tanks by sex, fed three times daily with live Artemia, and water quality was monitored. On the day before spawning, two females and four males were transferred to breeding tanks separated by transparent dividers; anti-predation inserts were used to prevent adults from eating the embryos. On the following morning, the dividers were removed and light was turned on to induce natural mating. Fertilized eggs were collected 1 h after spawning, rinsed with aquaculture water, and incubated in a constant temperature incubator at 28 °C. All zebrafish experiments were approved by the Animal Ethics Committee of the Nanjing normal university (Approval No. IACUC-2604002) and were conducted in accordance with relevant ethical guidelines.

### 2.3. Exposure Solution Preparation and Exposure Design

Based on preliminary experimental results, seven exposure groups were set for the acute toxicity test: 0 (CK), 0.1, 0.25, 0.5, 1, 2, and 4 mg/L TBBPA-MHEE. Based on the acute toxicity results and previously reported environmental levels, developmental and neurotoxicity tests were conducted at nominal concentrations of 0 (CK), 2 μg/L (environmentally relevant concentration), 20 μg/L (elevated concentration), and 200 μg/L (high sublethal concentration used for hazard characterization). Exposure solutions were prepared by serial dilution of a stock solution. Briefly, 10 mg of TBBPA-MHEE was accurately weighed and dissolved in 1 mL of DMSO to prepare a 10,000 mg/L stock solution; intermediate stock solutions were prepared by serial dilution, and working solutions were then prepared with embryo medium. For all treatment groups, DMSO was adjusted with vehicle solution so that the final DMSO volume fraction was 0.02% (*v*/*v*). The solvent control contained the same final DMSO concentration. In the recovery experiment, the concentration of TBBPA-MHEE was 200 μg/L and that of quercetin (QUE) was 3.5 mg/L [22]. The groups were designated as follows: CK (solvent control group), QUE (3.5 mg/L QUE group), MHEE (200 μg/L TBBPA-MHEE group), and MHEE+QUE (200 μg/L TBBPA-MHEE + 3.5 mg/L QUE group). In the quercetin intervention experiment, CK, QUE, MHEE, and MHEE+QUE groups were prepared with matched solvent backgrounds, and the final DMSO volume fraction was maintained at 0.02% (*v*/*v*) in all groups. After preparation, normally developing embryos at 4 h post-fertilization (hpf) were selected for exposure, which lasted for 6 days. Each treatment group consisted of three replicate wells, 5 mL exposure solution per well. In the acute toxicity test, 10 embryos were used per replicate well; in the developmental and neurotoxicity experiments, 20 embryos were used per replicate well. During exposure, water quality conditions were maintained as follows: temperature 27.5–28.5 °C, pH 7.0–8.0, and a photoperiod of 14 h light/10 h dark. Dead embryos were removed and recorded daily, and the exposure solution was renewed every 2 days. To obtain sufficient biological material for enzyme activity and gene expression analyses, a separate independent exposure experiment was conducted in this study. For each concentration group, 60 healthy embryos were randomly selected and placed in a 50 mL plastic round dish containing 30 mL of exposure solution, with six dishes per concentration (6 biological replicates). Of these, three dishes were allocated for enzyme activity assays and the other three for RT-qPCR analysis. All exposure conditions (temperature, photoperiod, etc.) were identical to those in the main experiment. In this study, wells or culture dishes were considered independent exposure units. Tubes were used only as sample-processing containers after exposure. For pooled biochemical and RT-qPCR assays, each tube contained larvae collected from one independently established exposure dish; therefore, each tube corresponded to one biological replicate derived from one exposure unit. Embryos were obtained from one spawning event, pooled after fertilization, and randomly allocated across treatment groups and replicate exposure units.

### 2.4. Image Acquisition and Phenotypic Analysis

Developmental and neural phenotypes of zebrafish larvae were imaged at different stages following TBBPA-MHEE exposure. A total of 15 zebrafish larvae were randomly selected from each treatment group (5 larvae per well, 3 biological replicates). At 24 and 72 hpf, videos of the heart region of zebrafish embryos and larvae were captured using a fluorescence stereomicroscope (Nikon SMZ25, Tokyo, Japan). The acquired videos were quantitatively analyzed using DanioScope v1.1 software (Noldus, Wageningen, The Netherlands) to measure the number of spontaneous movements at 24 hpf and the heart rate at 72 hpf. After 72 h and 144 h of exposure, images of different transgenic zebrafish lines were captured using a fluorescence stereomicroscope (Nikon SMZ25, Tokyo, Japan). NIS-Elements D v5.41.00 software was used to quantitatively analyze the pericardial area, body length, motor neuron synapse length, and GABAergic neuron fluorescence intensity at 72 hpf, as well as the body length, swim bladder area, eye area, motor neuron synapse length, and GABAergic neuron fluorescence intensity at 144 hpf. The fluorescence intensity quantification of GABAergic neurons was normalized to the mean fluorescence intensity of the control group. Prior to imaging, larvae that developed blood coagulation, death, severe spinal curvature, or pericardial edema before the endpoint measurement were excluded. During imaging, images with obvious blurring or those that did not completely cover the field of view were removed. We confirmed that each image was suitable for quantitative analysis. The number of independent exposure units, the number of larvae measured, the number of biological replicates for each endpoint, and the corresponding statistical test results are summarized in Table S2.

### 2.5. Locomotor Behavior Analysis

After 144 h of exposure, for each exposure group, 12 larvae were randomly selected (4 larvae per well, 3 biological replicates) and placed individually into 24-well plates (one larva per well). The plate was placed in an automated behavioral tracking system (Noldus, Netherlands) and allowed to acclimate for 5 min before video recording began. Recording lasted for a total of 21 min, including a 20 min light–dark transition test (5 min light/5 min dark, repeated twice), followed by a 1 min tapping stimulation test. During the tapping stimulation test, larvae were allowed to acclimate for 30 s, then stimulated twice with a 10 s interval between stimulations. After recording, the videos were quantitatively analyzed using EthoVision XT 16 software to quantify the following parameters: total swimming distance, average swimming speed, duration of immobility, average speed per minute during the light–dark transition, and the second-by-second speed response after tapping stimulation.

### 2.6. Protein–Protein Interaction (PPI) Network Analysis and Toxicity Prediction

The SMILES string of TBBPA-MHEE was obtained from the PubChem database (https://pubchem.ncbi.nlm.nih.gov/, accessed on 20 April 2026) and submitted to SwissTargetPrediction (https://swisstargetprediction.ch/, accessed on 20 April 2026) to predict potential molecular targets. Only targets with a probability score > 0 were retained for further analysis (default threshold of SwissTargetPrediction). Neurotoxicity-related genes were collected from the GeneCards database (https://www.genecards.org/, accessed on 20 April 2026) using “neurotoxicity” as the search term. Genes related to human diseases were selected using a relevance score threshold of ≥1. Overlapping genes between the predicted TBBPA-MHEE targets and the neurotoxicity-related genes were identified using the Draw Venn Diagram online tool (https://bioinformatics.psb.ugent.be/webtools/Venn/, accessed on 20 April 2026). The intersection targets were imported into the STRING database (https://cn.string-db.org/, accessed on 21 April 2026) to construct a protein–protein interaction network. A confidence score of ≥0.4 (medium confidence) was applied, and disconnected nodes were hidden. All target prediction and subsequent analyses in this study were directly performed using humans (*Homo sapiens*) as the species. The network file was exported in TSV format and visualized in Cytoscape software (v3.10.0), followed by node degree-based topological analysis. For structure-based toxicity prediction, the SMILES string of TBBPA-MHEE was uploaded to ProTox 3.0 to assess potential organ toxicity and relevant toxicological endpoints. We performed KEGG pathway enrichment analysis of the core targets using DAVID (https://davidbioinformatics.nih.gov/, accessed on 25 April 2026), employing the hypergeometric test with Benjamini–Hochberg false discovery rate correction. A corrected *p*-value < 0.05 was considered statistically significant.

### 2.7. ROS Staining and Enzyme Activity Assays

For ROS staining, 15 larvae (5 larvae per well, 3 biological replicates) were randomly selected from each treatment group after 144 h of TBBPA-MHEE exposure. The larvae were incubated with 10 μM 2′,7′-dichlorodihydrofluorescein diacetate (DCFH-DA, Beyotime, Shanghai, China) at 28 °C in the dark for 1 h. After washing three times with PBS (0.01 M, pH 7.4), images were acquired using a fluorescence stereomicroscope (Nikon SMZ25, Tokyo, Japan). The fluorescence intensity in the brain region of each larva was quantified using NIS-Elements D v5.41.00 software and normalized to the mean fluorescence intensity of the control group.

For enzyme activity assays, 150 larvae from each treatment group were collected (50 larvae per tube, 3 biological replicates) and homogenized by ultrasonic disruption in 200 μL of ice-cold saline (0.9% NaCl). Homogenates were centrifuged at 10,000× *g* for 15 min at 4 °C, and the supernatants were collected for biochemical measurements. Total protein concentration was determined using the BCA method (Beyotime, Shanghai, China), and the activities of superoxide dismutase (SOD) and catalase (CAT) were measured. Enzyme activities were normalized to the mean activity of the control group.

### 2.8. Real-Time Quantitative Polymerase Chain Reaction (RT-qPCR)

In this study, total RNA of zebrafish larvae was extracted using the TRIzol method. Three independent culture dishes (3 biological replicates) were set up for each treatment group. At 144 hpf, 50 larvae were randomly selected from each dish as one biological replicate sample, placed into 1.5 mL RNase-free microcentrifuge tubes, and homogenized with 1 mL of TRIzol reagent. Subsequently, chloroform and isopropanol were added sequentially for phase separation and RNA precipitation, followed by ethanol washing. The RNA pellet was dissolved in DEPC-treated water. RNA concentration and purity were measured using a Nanodrop 2000 spectrophotometer (Thermo Fisher Scientific, Waltham, MA, USA). cDNA was synthesized according to the instructions of the RT SuperMix kit. The quantitative PCR reaction mixture was prepared following the SYBR qPCR Master Mix kit instructions, and the reaction was performed on a CFX Connect™ Real-Time PCR Detection System (Bio-Rad, Hercules, CA, USA). The relative mRNA expression of target genes was normalized to β-actin and calculated using the 2^–ΔΔCt^ calculation method [19,23]. In this study, we did not perform standard curve validation for primer amplification efficiency in RT-qPCR analysis, nor did we employ algorithmic approaches to systematically evaluate the expression stability of the reference gene β-actin under different treatment conditions. These methodological shortcomings might, to some extent, affect the accuracy of the quantitative results. However, all amplification curves exhibited typical sigmoidal shapes, and the results were reproducible; therefore, these limitations are unlikely to alter the main conclusions of this study.

### 2.9. Statistics and Analysis of Data

All statistical analyses and graph plotting were performed using GraphPad Prism 8.0 software. All assays were performed with blinding to the experimental groups. Continuous variables are presented as mean ± standard error of the mean (SEM). A linear mixed-effects model was employed for statistical analysis, with treatment group fitted as a fixed factor and exposure well as a random factor to account for the non-independence of individual larval measurements obtained from the same well. Dunnett‘s test was applied for post hoc multiple comparisons, to compare each exposure group against the control group, as well as the quercetin rescue group against the TBBPA-MHEE-only exposure group. The statistical analyses of survival rate, hatching rate, enzyme activity and gene expression level were conducted using one-way ANOVA and Dunnett’s test. Statistical significance is indicated as follows: compared with the control group, * *p* < 0.05, ** *p <* 0.01, *** *p* < 0.001; for quercetin rescue experiments, comparisons between the MHEE and MHEE+QUE groups were indicated with # symbols, # *p* < 0.05, ## *p* < 0.01, ### *p* < 0.001.

## 3. Results

### 3.1. TBBPA-MHEE Exposure Induces Developmental Toxicity in Zebrafish Larvae

**Figure 1:**
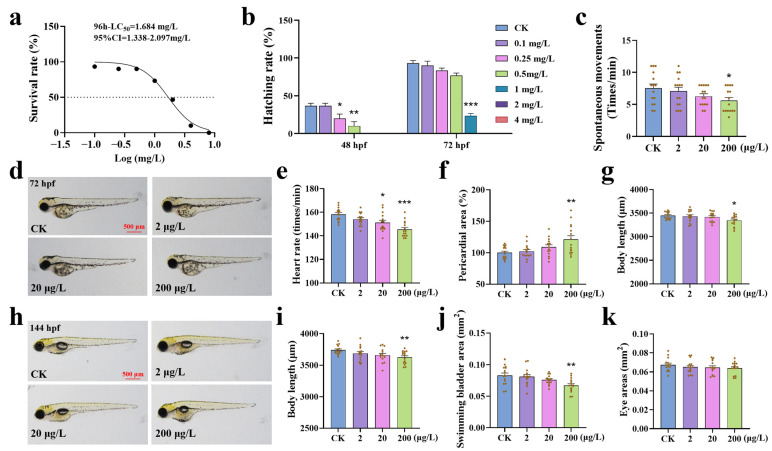
TBBPA-MHEE exposure induces developmental toxicity in zebrafish larvae. (**a**) Survival curve of zebrafish embryos/larvae after TBBPA-MHEE exposure and the 96 h LC_50_, n = 3; (**b**) hatching rates at 48 and 72 hpf under different exposure concentrations, n = 3; (**c**) spontaneous movement frequency at 24 hpf, n = 15; (**d**) representative images of larvae at 72 hpf and quantitative analysis of (**e**) heart rate, (**f**) pericardial area, and (**g**) body length, n = 15; (**h**) representative images of larvae at 144 hpf and quantitative analysis of (**i**) body length, (**j**) swim bladder area, and (**k**) eye area, n = 15. Data are presented as mean ± SEM. * *p* < 0.05, ** *p* < 0.01, *** *p* < 0.001.

The acute toxicity test results showed that the 96 h median lethal concentration (96 h-LC_50_) of TBBPA-MHEE for zebrafish embryos/larvae was 1.684 mg/L, with a 95% confidence interval of 1.338–2.097 mg/L (Figure 1a). Moreover, TBBPA-MHEE at nominal concentrations of 0.25 mg/L and above significantly inhibited embryonic hatching (Figure 1b, *p* < 0.001). In the developmental toxicity test, compared with the control group, the spontaneous movement frequency at 24 hpf was significantly reduced in the 200 μg/L exposure group (Figure 1c, *p* < 0.05). At 72 hpf (Figure 1d), compared with the control group, the heart rate of larvae in the 20 and 200 μg/L TBBPA-MHEE exposure groups was significantly decreased (Figure 1e, *p* < 0.001). The 200 μg/L group also exhibited a significantly enlarged pericardial area (Figure 1f, *p* < 0.01) and a significantly shortened body length (Figure 1g, *p* < 0.05). At 144 hpf (Figure 1h), compared with the control group, larvae exposed to 200 μg/L TBBPA-MHEE showed significantly reduced body length and swim bladder area (Figure 1i,j, *p* < 0.01), whereas no significant difference was observed in eye area.

### 3.2. TBBPA-MHEE Exposure Impairs Locomotor Behavior in Larvae

**Figure 2:**
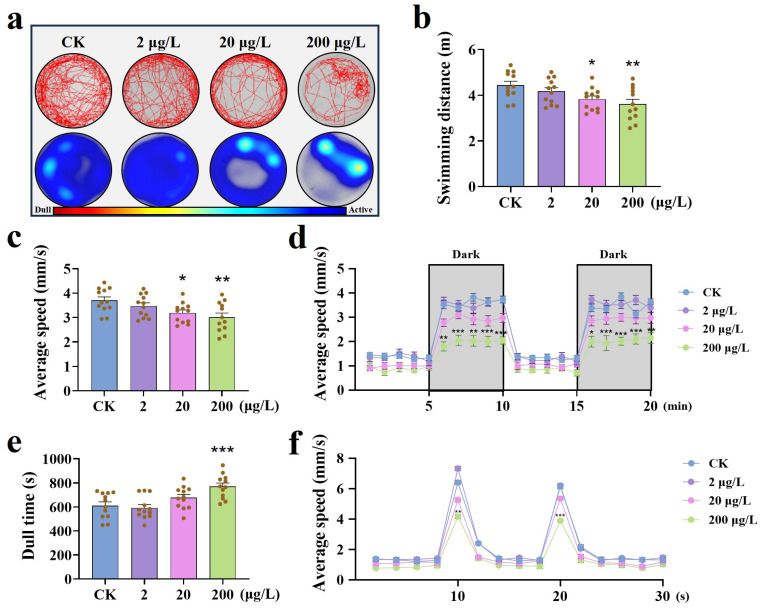
TBBPA-MHEE exposure impairs locomotor behavior in larvae. (**a**) Locomotor trajectories (upper) and heatmaps (lower) of zebrafish larvae after 144 h of TBBPA-MHEE exposure, n = 12; (**b**) total swimming distance, n = 12; (**c**) average swimming speed, n = 12; (**d**) changes in swimming speed under light–dark transition stimulation, n = 12; (**e**) immobility time, n = 12; (**f**) swimming speed in response to tapping test, n = 12. Data are presented as mean ± SEM. * *p* < 0.05, ** *p* < 0.01, *** *p* < 0.001.

To further evaluate the neurobehavioral consequences of sublethal TBBPA-MHEE exposure, the locomotor behavior of larvae at 144 hpf from different nominal exposure concentration groups was analyzed. The results showed that control larvae exhibited widespread swimming trajectories and high overall activity levels, whereas with increasing nominal exposure concentrations, the locomotor trajectories became progressively restricted, and activity levels decreased (Figure 2a). Quantitative analysis indicated that the swimming distance and swimming speed of larvae in the 20 and 200 μg/L TBBPA-MHEE exposure groups were significantly reduced (Figure 2b,c, *p* < 0.01). In the light–dark transition test, the increase in swimming speed during the dark phase was attenuated in the 200 μg/L group, and the speed was significantly reduced compared with the control group (Figure 2d, *p* < 0.05), indicating impaired responsiveness to light changes. Following exposure to 200 μg/L TBBPA-MHEE, the duration of immobility was significantly prolonged (Figure 2e, *p* < 0.01), and the speed response induced by tapping stimulation was also reduced (Figure 2f), further confirming impaired responsiveness to external stimuli.

### 3.3. TBBPA-MHEE Exposure Impairs Neurodevelopment in Zebrafish Larvae

**Figure 3:**
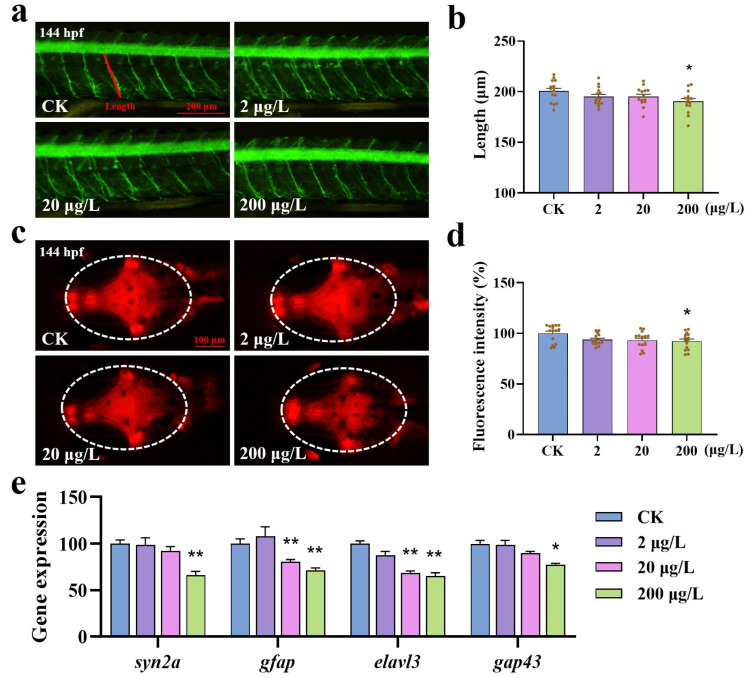
Neurotoxicity of TBBPA-MHEE exposure in zebrafish larvae. (**a**) Fluorescence images of motor neurons in *Tg(hb9:eGFP)* zebrafish larvae at 144 hpf and (**b**) quantitative analysis of motor neuron synapse length, n = 15; (**c**) fluorescence images of head neurons in *Tg(gad1b:mCherry)* zebrafish larvae at 144 hpf and (**d**) quantitative analysis of relative fluorescence intensity, n = 15, the white circle represents the quantitative analysis area for fluorescence intensity; (**e**) expression levels of neurodevelopment-related genes (*syn2a*, *gfap*, *elavl3*, *gap43*), n = 3. Data are presented as mean ± SEM. * *p* < 0.05, ** *p* < 0.01.

The effects of TBBPA-MHEE on neurodevelopment were further investigated using *Tg(hb9:eGFP)* and *Tg(gad1b:mCherry)* transgenic zebrafish. The results showed that compared with the control group, the motor neuron synapse length of *Tg(hb9:eGFP)* larvae was significantly reduced in the 200 μg/L TBBPA-MHEE exposure group (Figure 3a,b, *p* < 0.05), and the fluorescence intensity of central neurons in *Tg(gad1b:mCherry)* larvae was significantly attenuated (Figure 3c,d, *p* < 0.05). Moreover, the expression levels of early neurodevelopment-related genes, including *syn2a*, *gfap*, *elavl3*, and *gap43*, showed an overall decreasing trend in the TBBPA-MHEE exposure groups, with significant reductions observed for some genes. These results indicate that TBBPA-MHEE exposure may interfere with the development of motor neurons and GABAergic neurons in zebrafish larvae.

### 3.4. Candidate Targets and Pathways Associated with TBBPA-MHEE-Induced Neurotoxicity

**Figure 4:**
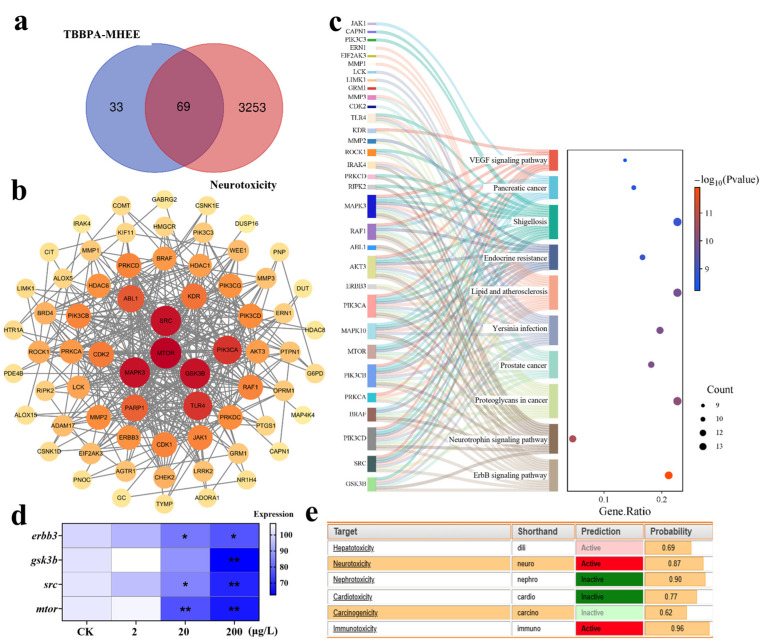
Potential targets and pathways of TBBPA-MHEE-induced neurotoxicity in zebrafish larvae. (**a**) Venn diagram of TBBPA-MHEE-related targets and neurotoxicity targets; (**b**) protein–protein interaction (PPI) network of intersecting targets, where darker and larger nodes indicate higher degree centrality; (**c**) KEGG pathway enrichment analysis of intersecting targets, showing target gene list on the left, pathway connections in the middle, and enrichment bubble plot on the right; (**d**) heatmap of expression of key core genes (*erbb3*, *gsk3b*, *src*, *mtor*), n = 3; (**e**) toxicity endpoint prediction results based on the structure of TBBPA-MHEE. Data are presented as mean ± SEM. * *p* < 0.05, ** *p* < 0.01.

To explore the potential molecular mechanisms underlying TBBPA-MHEE-induced neurotoxicity in zebrafish, network toxicology analysis was performed. A total of 69 intersecting targets were identified between the predicted TBBPA-MHEE-related targets and neurotoxicity-associated genes (Figure 4a). A PPI network was constructed based on these intersecting genes, and the analysis showed that MTOR, SRC, MAPK3, and GSK3B had high degree centrality, suggesting they might be central nodes of the toxicity network (Figure 4b). KEGG enrichment analysis further identified the ErbB signaling pathway as one of the significantly enriched pathways, with key genes such as erbb3, gsk3b, src, and mtor being involved in or closely associated with this pathway (Figure 4c). RT-qPCR analysis showed that the expression levels of erbb3, gsk3b, src, and mtor were significantly decreased after TBBPA-MHEE exposure (Figure 4d, *p* < 0.01). Additionally, structure-based prediction using ProTox 3.0 suggested that TBBPA-MHEE may have relatively high probabilities of neurotoxicity and immunotoxicity, reaching 0.87 and 0.96, respectively (Figure 4e).

### 3.5. TBBPA-MHEE Exposure Induces Oxidative Stress, Inflammation, and Apoptotic Responses

**Figure 5:**
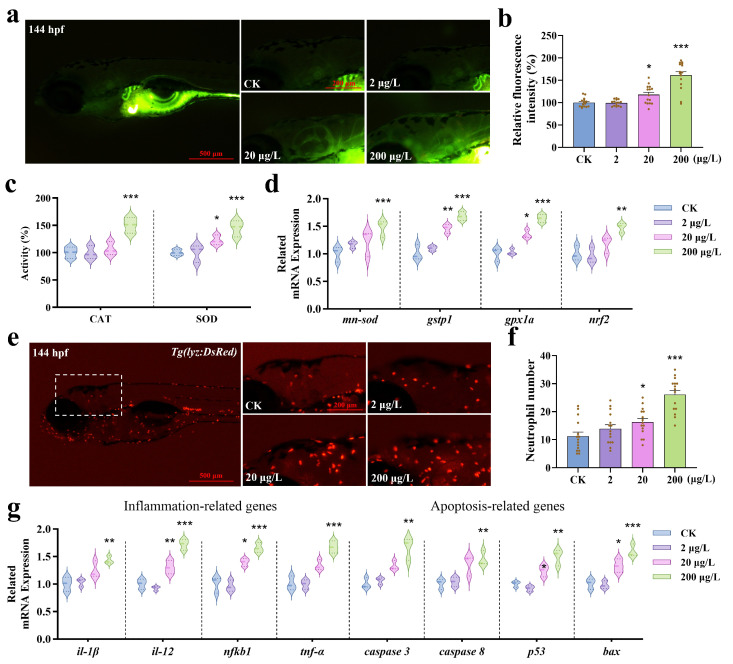
TBBPA-MHEE induces oxidative stress, inflammation, and apoptosis in zebrafish. (**a**) Representative images of reactive oxygen species (ROS) fluorescence staining in zebrafish larvae at 144 hpf and (**b**) quantitative analysis of relative fluorescence intensity, n = 15; (**c**) changes in CAT and SOD activities, n = 3; (**d**) quantitative analysis of oxidative stress-related gene expression (*mn-sod*, *gstp1*, *gpx1a*, *nrf2*), n = 3; (**e**) representative images of neutrophil infiltration in the brain of *Tg(lyz:DsRed)* transgenic zebrafish and (**f**) quantitative analysis of neutrophil number, n = 15, the white dashed box represents the area for quantitative analysis of inflammatory factors in the brain of larvae; (**g**) quantitative analysis of inflammation-related gene expression (*il-1β*, *il-12*, *nfkb1*, *tnf-α*) and apoptosis-related gene expression (*caspase 3*, *caspase 8*, *p53*, *bax*), n = 3. Data are presented as mean ± SEM. * *p* < 0.05, ** *p* < 0.01, *** *p* < 0.001.

To further investigate the oxidative stress and inflammatory responses in zebrafish following TBBPA-MHEE exposure, this study combined ROS fluorescence staining, antioxidant enzyme activity assays, RT-qPCR, and *(lyz:DsRed)* transgenic zebrafish. ROS staining results showed that compared with the control group, the relative fluorescence intensity of ROS in the brain region of larvae in the 20 μg/L and 200 μg/L TBBPA-MHEE exposure groups was significantly increased (Figure 5a,b, *p* < 0.05). Enzyme activity assays and qPCR results demonstrated that the activities of SOD and CAT, as well as the expression levels of oxidative stress-related genes (*mn-sod*, *gstp1*, *gpx1a*, and *nrf2*), were significantly elevated (Figure 5c,d, *p* < 0.05). Furthermore, compared with the control group, the number of neutrophils in the brain region of *Tg(lyz:DsRed)* larvae was significantly increased in the 20 and 200 μg/L TBBPA-MHEE exposure groups (Figure 5e,f, *p* < 0.05). Meanwhile, the expression levels of inflammation-related genes (*il-1β*, *il-12*, *nfkb1*, and *tnf-α*) and apoptosis-related genes (*caspase 3*, *caspase 8*, *p53*, and *bax*) were significantly upregulated. These results indicate that TBBPA-MHEE exposure induces oxidative stress and inflammatory responses, accompanied by altered expression of apoptosis-related genes.

### 3.6. Quercetin Partially Alleviates TBBPA-MHEE-Induced Oxidative/Inflammatory Responses and Selected Developmental and Behavioral Phenotypes

**Figure 6:**
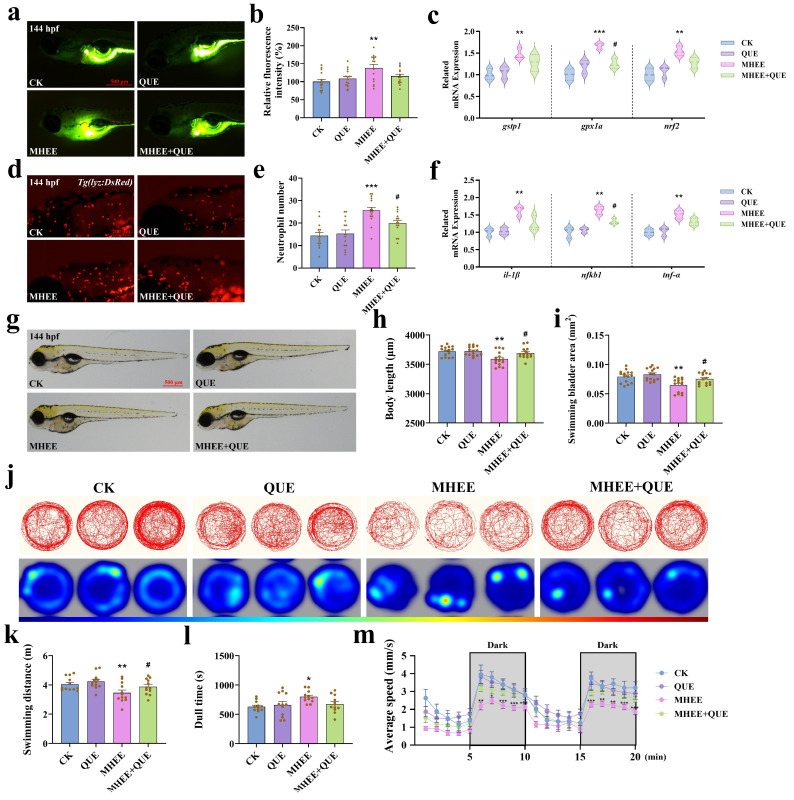
Quercetin alleviates neurodevelopmental toxicity in zebrafish through antioxidant and anti-inflammatory effects. (**a**) ROS fluorescence staining images of zebrafish larvae at 144 hpf and (**b**) quantitative analysis of relative fluorescence intensity, n = 15; (**c**) quantitative analysis of oxidative stress-related gene expression (*gsp1*, *gpx1a*, *nrf2).* n = 3; (**d**) representative images of neutrophil infiltration in *Tg(lyz:DsRed)* transgenic zebrafish and (**e**) quantitative analysis of neutrophil number, n = 15; (**f**) quantitative analysis of inflammation-related gene expression (*il-1β*, *nfkb1*, *tnf-α*), n = 3; (**g**) representative images of zebrafish larvae at 144 hpf and quantitative analysis of (**h**) body length and (**i**) swim bladder area, n = 15; (**j**) locomotor trajectories (upper) and heatmaps (lower) of zebrafish larvae; (**k**) total swimming distance, (**l**) immobility time, and (**m**) swimming speed under light–dark transition stimulation, n = 12. Data are presented as mean ± SEM. * *p* < 0.05, ** *p* < 0.01, *** *p* < 0.001 indicate significant differences compared with the control group; # *p* < 0.05 indicates significant difference between the quercetin rescue group and the MHEE group.

To further verify the roles of oxidative stress and inflammation in TBBPA-MHEE-induced neurodevelopmental toxicity, a quercetin rescue experiment was conducted. Compared with the TBBPA-MHEE-only exposure group, quercetin treatment reduced the relative ROS fluorescence intensity in the larval brain region (Figure 6a,b). The expression levels of oxidative stress-related genes *gstp1*, *gpx1a*, and *nrf2* showed an overall decreasing trend, with a significant reduction observed for gpx1a (Figure 6c, *p* < 0.05). Meanwhile, the number of neutrophils in the larval brain region was significantly decreased in the quercetin intervention group (Figure 6d,e, *p* < 0.05), and the expression levels of inflammation-related genes *il-1β*, *nfkb1*, and *tnf-α* were also markedly downregulated (Figure 6f, *p* < 0.05). In terms of developmental phenotypes, compared with the TBBPA-MHEE-exposed group, larvae in the quercetin rescue group exhibited significantly increased body length and swim bladder area (Figure 6g–i, *p* < 0.05). Behavioral analysis further showed that quercetin significantly increased the total swimming distance of larvae (Figure 6k, *p* < 0.05), decreased the duration of immobility, and partially restored the speed response during the light–dark transition (Figure 6l,m). These findings indicate that quercetin can partially alleviate TBBPA-MHEE-induced oxidative stress, inflammation, and the associated developmental and behavioral abnormalities.

## 4. Discussion

With the continuous production and application of TBBPA-based flame retardants, the ecological risks posed by their byproducts (including TBBPA-MHEE) have attracted increasing concern. However, studies on the developmental and neurotoxic effects of TBBPA-MHEE in whole organisms remain very limited. In this study, using zebrafish at early developmental stages as a model, we systematically evaluated the neurodevelopmental toxicity of TBBPA-MHEE across multiple biological levels, including individual development, locomotor behavior, neural structural phenotypes, molecular signaling, and intervention validation.

This study demonstrates for the first time that TBBPA-MHEE has clear early developmental toxicity. Acute toxicity tests showed that exposure to relatively high nominal concentrations of TBBPA-MHEE reduced survival rates and inhibited embryonic hatching. At sublethal nominal concentrations, larvae exhibited reduced spontaneous embryonic movement, shortened body length, pericardial edema, and impaired swim bladder development, indicating concentration-dependent developmental interference. These findings are consistent with previous reports that TBBPA-related flame retardants and their structural analogs induce developmental toxicity in zebrafish [3,24]. The swim bladder is critical for maintaining buoyancy, posture, and efficient swimming in zebrafish [25]. Therefore, impaired swim bladder development may directly contribute to reduced locomotor ability [26]. Behavioral test results showed that TBBPA-MHEE exposure significantly reduced total swimming distance and average swimming speed, prolonged immobility duration, and attenuated larval responses to light–dark transition and tapping stimulation. Spontaneous movement, light–dark response, and escape-like behavior are widely used as sensitive functional endpoints for neurotoxicity assessment in zebrafish [27]. Environmental pollutants can interfere with neurodevelopment through processes such as oxidative stress, inflammatory responses, and apoptosis, ultimately leading to behavioral dysfunction [28,29]. In this study, larvae in the 200 μg/L group exhibited shortened body length, increased incidence of pericardial edema, reduced heart rate, impaired swim bladder development, and decreased swimming activity, At the nominal concentration of 20 μg/L, behavioral changes were observed together with a significant reduction in heart rate. Therefore, these behavioral effects should not be interpreted as evidence of primary neurotoxicity alone, but may reflect combined neural, cardiac, developmental, buoyancy-related, and general physiological effects.

Direct neurobehavioral phenotypic changes were also observed in this study. Motor neurons coordinate swimming behavior by precisely regulating the timing and intensity of muscle contractions [30,31]. In *Tg(hb9:eGFP)* larvae, TBBPA-MHEE exposure significantly shortened motor neuron synapse development. Furthermore, gad1b is closely related to the synthesis of GABAergic neurotransmitters and the regulation of neural function in zebrafish, and its expression status is associated with the excitation–inhibition balance of the central nervous system and locomotor behavior [32,33]. The reduced fluorescence signal in the brain region observed in *Tg(gad1b:mCherry)* larvae, along with decreased expression of neurodevelopment-related genes *syn2a*, *gfap*, *elavl3*, and *gap43*, indicates that TBBPA-MHEE exposure may impair the development of the central nervous system and motor neuron synapses in larvae.

At the mechanistic level, network toxicology analysis predicted potential molecular targets of TBBPA-MHEE-induced neurotoxicity. Intersection analysis and PPI network construction revealed that MTOR, SRC, MAPK3, and GSK3B are nodes with a relatively high degree centrality, suggesting their involvement in the potential toxicity network. KEGG enrichment analysis further identified the ErbB signaling pathway as one of the significantly enriched pathways. The ErbB signaling pathway is involved in the proliferation, migration, and survival of neural cells [34,35,36] as well as neuronal differentiation [37]. Dysregulation of this pathway is associated with abnormal brain development, altered embryonic movement, and impaired motor neuron development [38,39]. Notably, mTOR is not only a key effector of the PI3K/Akt/mTOR signaling axis but also an important regulator of neuronal growth and axon development [40,41]. Previous studies have demonstrated that impaired locomotor function in zebrafish is associated with dysregulation of the PI3K/Akt/mTOR axis [42]. In addition, GSK3B and SRC are involved in downstream and upstream regulatory processes of this signaling network, respectively [43,44,45], and ErbB signaling can functionally interact with the PI3K/Akt/mTOR axis [46,47]. Combined with the observed downregulation of *erbb3*, *gsk3b*, *src*, and *mtor* expression in this study, these results suggest that TBBPA-MHEE may interfere with the ErbB-related signaling network, thereby affecting PI3K/Akt/mTOR-related molecular responses and inducing neurotoxicity in zebrafish.

Oxidative stress, inflammatory responses, and apoptosis may be important downstream processes linking signaling pathway dysregulation to neurotoxic phenotypes. Previous studies have shown that reduced PI3K/Akt/mTOR activity is associated with enhanced inflammation, neuronal damage, and apoptosis [48,49,50], whereas activation of this signaling axis alleviates ROS accumulation, inflammation, and cell death [51,52]. In this study, TBBPA-MHEE exposure led to increased ROS fluorescence in the larval brain, elevated CAT and SOD activities, and upregulation of oxidative stress-related genes *mn-sod*, *gstp1*, *gpx1a*, and *nrf2*. At the same time, neutrophil recruitment in the brain region was increased, and inflammation-related genes *il-1β*, *il-12*, *nfkb1*, and *tnf-α* as well as apoptosis-related genes *caspase 3*, *caspase 8*, *p53*, and *bax* were significantly upregulated. These results indicate that TBBPA-MHEE exposure induces oxidative stress and induce changes in the expression levels of genes related to inflammation and apoptosis. Studies have shown that TBBPA exposure can induce developmental abnormalities, oxidative stress, and apoptosis-related changes in zebrafish embryos and larvae [53], which is generally consistent with our findings. To further evaluate the roles of oxidative stress and inflammation, we conducted a rescue experiment using quercetin. Quercetin is a flavonoid with well-defined antioxidant and anti-inflammatory properties [22,54]. Previous studies have reported that quercetin alleviates oxidative stress and neuroinflammation and improves neurobehavioral phenotypes in zebrafish. The quercetin concentration was selected based on previous zebrafish studies reporting protective effects of quercetin against oxidative stress, inflammation, or developmental toxicity [55,56,57]. In this study, quercetin treatment reduced ROS accumulation and neutrophil recruitment, suppressed the induction of inflammatory genes, and partially restored body length, swim bladder development, and locomotor behavior, consistent with previous studies. These results further support that oxidative stress and inflammatory responses are involved in TBBPA-MHEE-induced neurodevelopmental toxicity.

This study has several limitations. First, the mechanistic interpretation is primarily based on network toxicology predictions, RT-qPCR validation, and quercetin rescue experiments. Direct evidence from protein, phosphorylation, or pathway perturbation levels is still needed to establish a causal relationship between TBBPA-MHEE exposure and the ErbB-PI3K/Akt/mTOR axis. A key limitation of this study is that we did not perform pathway-specific rescue experiments using pharmacological activators (e.g., IGF-1 for PI3K/Akt or MHY1485 for mTOR). Therefore, we cannot establish a causal role of this pathway in TBBPA-MHEE-induced neurotoxicity. This important question remains a priority for future investigation. Second, although the nominal exposure concentration design in this study covered reported environmental detection levels and extended to higher concentrations for hazard characterization, future studies integrating measured environmental exposure concentrations, in vivo accumulation, and chronic low-dose exposure scenarios that reflect real-world conditions are needed to improve ecological risk extrapolation. Moreover, adsorption, degradation, or loss that may occur during the renewal process can lead to differences between actual exposure concentrations and nominal values. Exposure-medium concentrations and stock-solution stability were not analytically verified. Therefore, this study is now defined as a nominal concentration hazard screening and mechanistic hypothesis study rather than a definitive exposure–response assessment. Because quercetin is biologically pleiotropic, these results support the involvement of oxidative stress and inflammation but do not establish these responses as the sole upstream cause of TBBPA-MHEE-induced toxicity. Finally, the metabolic transformation, toxicokinetics, and mixture toxicity of TBBPA-MHEE together with its parent compound or other derivatives have not yet been explored and warrant further investigation.

In summary, this study provides the first systematic in vivo assessment of TBBPA-MHEE-induced neurodevelopmental toxicity in zebrafish. By integrating developmental phenotyping, behavioral analysis, transgenic neuro zebrafish models, network toxicology, molecular detection, and quercetin rescue experiments, we propose a potential mode of action: TBBPA-MHEE exposure interferes with the ErbB-related signaling network and PI3K/Akt/mTOR-related molecular responses, accompanied by the occurrence of oxidative stress, inflammation, and apoptotic events, ultimately impairing early neurodevelopment and locomotor behavior in zebrafish larvae. These findings provide experimental evidence for the toxicity identification and ecological risk assessment of TBBPA-derived pollutants.

## 5. Conclusions

In conclusion, nominal sublethal exposure to TBBPA-MHEE induced developmental abnormalities, locomotor deficits, and neural injury-related phenotypes in zebrafish larvae. These effects were accompanied by oxidative stress, inflammatory responses, apoptosis-related-associated signals, and altered transcription of genes associated with ErbB and PI3K/Akt/mTOR-related signaling. Quercetin partially alleviated oxidative/inflammatory responses and improved selected phenotypic outcomes, supporting the involvement of oxidative stress and inflammation. Further studies with measured exposure concentrations, internal dose assessment, protein/phosphorylation-level validation, and pathway-specific perturbation are needed to confirm causal mechanisms.

## Data Availability

The raw data supporting the conclusions of this article will be made available by the authors upon request.

## Supplementary Materials

The following supporting information can be downloaded at https://www.mdpi.com/article/10.3390/biology15141120/s1, Table S1: Sequences of primers for the genes tested; Table S2: Experimental design and statistical details for all endpoints and figures.
