# Supplementary material for: Developmental and Neurobehavioral Toxicity of Tetrabromobisphenol A Mono(2-hydroxyethyl) Ether (TBBPA-MHEE) in Zebrafish Larvae: Oxidative/Inflammatory Responses and Candidate ErbB-Related Signaling
Source: Biology (Basel). 2026 Jul 10;15(14):1120. doi: 10.3390/biology15141120 (PMC13403645; doi:10.3390/biology15141120)
Supplement: Supplementary file 1 [file biology-15-01120-s001.zip › biology-4366126-supplementary.pdf]

Supplementary materials

Table S1 Sequences of primers for the genes tested

Gene	Forward (5'-3')	Reverse (5'-3')
β-actin	TCTGGCATCACACCTTCTACAAT	TGTTGGCTTTGGGATTCAGG
mn-sod	AATCAGCAGGTTCTTCGGAGGAGA	ACACTCGGTTGCTCTCTTTTCTCT
gstp1	AGGCTTCCCTCATTGACGTG	TCCACCCAAGGTCTGTGAGA
gpx1a	GAAGCCATTTCCAGGACGGA	ATTTGGGTTGCCCAACAGGA
nrf2	AAGCAGACGGAGGAGGAG	GGAGGTGTTCAGGCAAGG
il-1β	ATGGCGAACGTCATCCAAGA	GAGACCCGCTGATCTCCTTG
il-12	TCCATCACCGTCAACAGAGC	ATGCGGTGGTGTAGTGAGTG
nfkb1	GACCTGAACCTCCAGAAGCC	ACCTGCAGTGTTGTAGACCG
tnf-α	GCTGGATCTTCAAAGTCGGGTGTA	TGTGAGTCTCAGCACACTTCCATC
caspase 3	GAGACCGCTGCCCATCACTAG	ATCCTTTCACGACCATCT
caspase 8	AAGACCTGATTCTGCGACTG	TAGGCTGAGACACCTTTACG
p53	CCCGGATGGAGATAACTTG	CACAGTTGTCCATTCAGCAC
bax	TCGAACATGATCTGTGTCATC	TATGGCTGGGGTCACTTTTCTC
mtor	GTTTTCACTGTCCGTTGGGC	TGCTGGTGTGCCTAAAGCAT
erbb3	TTTGGGTCCGGGTCAGAATG	TTTCTACACACACCTCTCCGC
gsk3b	TGCCACTCTGTACACGGTTT	TGACGAAATCCTGGAGCGTC
src	GGGTTTGACTCCCTTCTGCC	ACCAGTCACCCTCCGTGTTA

Table S2 Experimental design and statistical details for all endpoints and figures

Figure	Endpoint	Exposure experiment	Larvae Number	Exposure units	Statistical unit	Data points shown
Figure 1	Suivival rate	acute toxicity	30	well	ANOVA	-
	Hatching rate	acute toxicity	30	well	ANOVA	-
	Spontaneous movement	Developmental toxicity	15	well	Mixed model	larvae
	Heart rate	Developmental toxicity	15	well	Mixed model	larvae
	Pericardial area	Developmental toxicity	15	well	Mixed model	larvae
	72 hpf Body length	Developmental toxicity	15	well	Mixed model	larvae
	144 hpf Body length	Developmental toxicity	15	well	Mixed model	larvae
	Swimming bladder area	Developmental toxicity	15	well	Mixed model	larvae
	Eye area	Developmental toxicity	15	well	Mixed model	larvae
Figure 2	Swimming distance	Locomotor behavior	12	well	Mixed model	larvae
	Average speed	Locomotor behavior	12	well	Mixed model	larvae
	Average speed under light/dark transition	Locomotor behavior	12	well	Mixed model	larvae
	Dull time	Locomotor behavior	12	well	Mixed model	larvae

	Average speed in response to tap stimulus	Locomotor behavior	12	well	Mixed model	larvae
**Figure 3**	length of motor nerve synapses	Neurotoxicity	15	well	Mixed model	larvae
	Neuronal fluorescence intensity	Neurotoxicity	15	well	Mixed model	larvae
	Gene expression	Neurotoxicity	150	dish/tube	ANOVA	-
**Figure 4**	Pathway related Gene expression	Mechanism Exploration	150	dish/tube	ANOVA	-
**Figure 5**	ROS fluorescence intensity	Mechanism Exploration	15	well	Mixed model	larvae
	enzymatic activity	Mechanism Exploration	150	dish/tube	ANOVA	-
	Oxidative stress related Gene expression	Mechanism Exploration	150	dish/tube	ANOVA	-
	Neutrophil number	Mechanism Exploration	15	well	Mixed model	larvae
	Inflammation and apoptosis related Gene expression	Mechanism Exploration	150	dish/tube	ANOVA	-
**Figure 6**	ROS fluorescence intensity	Recovery test	15	well	Mixed model	larvae
	Oxidative stress related Gene expression	Recovery test	150	dish/tube	ANOVA	-
	Neutrophil number	Recovery test	15	well	Mixed model	larvae
	Inflammation related Gene expression	Recovery test	150	dish/tube	ANOVA	-
	144 hpf Body length	Recovery test	15	well	Mixed model	larvae

	Swimming bladder area	Recovery test	15	well	Mixed model	larvae
	Swimming distance	Recovery test	12	well	Mixed model	larvae
	Average speed under light/dark transition	Recovery test	12	well	Mixed model	larvae
	Dull time	Recovery test	12	well	Mixed model	larvae
